# Cost-Effectiveness Analysis of *Helicobacter pylori* Diagnostic Methods in Patients with Atrophic Gastritis

**DOI:** 10.1155/2017/2453254

**Published:** 2017-02-23

**Authors:** Fumio Omata, Takuro Shimbo, Sachiko Ohde, Gautam A. Deshpande, Tsuguya Fukui

**Affiliations:** ^1^Department of Internal Medicine, St. Luke's International Hospital, Chuo-Ku, Japan; ^2^Center for Clinical Epidemiology, St. Luke's International University, Chuo-Ku, Japan; ^3^Ohta Nishinouchi Hospital, Koriyama, Japan

## Abstract

*Background*. There are several diagnostic methods for *Helicobacter pylori (H. pylori)* infection. A cost-effective analysis is needed to decide on the optimal diagnostic method. The aim of this study was to determine a cost-effective diagnostic method in patients with atrophic gastritis (AG). *Methods*. A decision-analysis model including seven diagnostic methods was constructed for patients with AG diagnosed by esophagogastroduodenoscopy. Expected values of cost and effectiveness were calculated for each test. *Results*. If the prevalence of *H. pylori* in the patients with AG is 85% and CAM-resistant *H. pylori* is 30%, histology, stool *H. pylori* antigen (SHPAg), bacterial culture (BC), and urine *H. pylori* antibody (UHPAb) were dominated by serum *H. pylori* IgG antibody (SHPAb), rapid urease test (RUT), and urea breath test (UBT). Among three undominated methods, the incremental cost-effective ratios (ICER) of RUT versus SHPAb and UBT versus RUT were $214 and $1914, respectively. If the prevalence of CAM-sensitive *H. pylori* was less than 55%, BC was not dominated, but its *H. pylori* eradication success rate was 0.86. *Conclusions*. RUT was the most cost-effective at the current prevalence of CAM-resistant *H. pylori*. BC could not be selected due to its poor effectiveness even if CAM-resistant *H. pylori* was more than 45%.

## 1. Introduction

While the prevalence of *Helicobacter pylori (H. pylori)* has been decreasing [1, 2], it remains a critical public health issue. Recently, increasing prevalence of CAM-resistant *H. pylori* is an emerging problem of public health all over the world as CAM is included in most first-line empiric *H. pylori* eradication regimens [3, 4].

Since the discovery of *H. pylori*, its association with peptic ulcer disease (PUD) [5], atrophic gastritis (AG) [6], gastric cancer [7], mucosa-associated lymphoid tissue (MALT) lymphoma [8], and immune thrombocytopenia [9] has been elucidated. Accordingly, the indication of *H. pylori* eradication therapy has been broadened from only PUD to some of the above diseases.

Among these *H. pylori*-related diseases, AG is more common than PUD or early gastric cancer; in Japan, its prevalence is reported to be approximately 27.9% even in healthy individuals [10]. 85% of AG patients were reported to have *H. pylori* infection [11]. It is a common situation that, during either diagnostic or screening esophagogastroduodenoscopy (EGD), physicians must choose between one of several *H. pylori* diagnostic methods.

There are three invasive methods to diagnose *H. pylori* infection during EGD, including rapid urease test (RUT), histology, and bacterial culture (BC) from biopsy specimens. Other noninvasive options to diagnose *H. pylori* are serum *H. pylori* IgG antibody (SHPAb), urea breath test (UBT), stool *H. pylori* antigen (SHPAg), and urine *H. pylori* IgG antibody (UHPAb).

The diagnostic performance of these tests differs. Using BC for diagnosing *H. pylori* infection allows us to perform antibiotic- (typically macrolide-) sensitivity testing. The results of the sensitivity testing are useful to make appropriate decisions when choosing the correct first regimen for treatment, a strategy called antimicrobial susceptibility-guided therapy (AMSGT). AMSGT is assumed to be more cost-effective when the prevalence of CAM-resistant *H. pylori* has been increasing. However, there have been no prior reports mainly focusing on the impact of the prevalence of CAM-resistant *H. pylori* infection. The aim of this study was to determine a cost-effective diagnostic method for *H. pylori* infection in patients with AG.

## 2. Methods

This study was conducted from a social perspective. A decision-analysis model was constructed for patients in Japan diagnosed with AG suggesting *H. pylori* infection, using screening or diagnostic EGD. Time horizon was until successful *H. pylori* eradication or the end of the third regimen. We assumed that this time horizon would fall within 1 year and did not discount either effectiveness or cost.

Undergoing one of seven diagnostic tests (RUT, histology, BC, SHPAb, UBT, SHPAg, and UHPAb), patients' *H. pylori* infection status was unknown. Excluding BC which can be applied for AMSGT, if one of six tests (RUT, histology, SHPAb, UBT, SHPAg, and UHPAb) was selected and was positive, the patient underwent empiric antibiotic treatment, as none of these six tests provided any information on CAM-sensitivity. If the first standard regimen failed, patients followed the second and third regimens without additional CAM-sensitivity testing. If BC was initially selected as the diagnostic test and was positive, subsequent antibiotic susceptibility testing results were used to decide on the treatment regimen. If detected *H. pylori* was sensitive to CAM, these patients were treated with CAM-included regimen. If not, these patients were treated with metronidazole-included regimen. In the decision tree, all eradication failure was measured by UBT after the previous diagnostic step. We did not include the strategy of initial six diagnostic tests followed by AMSGT as BC required repeat EGD and we considered it unaffordable to perform repeat EGD only for the purpose of BC.

Diagnostic performance, including sensitivity and specificity of invasive and noninvasive diagnostic tests, was obtained from past English literatures, searched manually through MEDLINE and EMBASE. If there was a literature of meta-analysis, we adopted pooled values of sensitivity and specificity. Otherwise, we conducted a meta-analysis (bivariate random effects model) to calculate pooled values of sensitivity and specificity. Effectiveness was measured by rate of successful *H. pylori* eradication.

We used the success rate of *H. pylori* eradication by the first regimen (lansoprazole 30 mg bid, amoxicillin 750 mg or 1000 mg bid, and CAM 200 mg or 500 mg bid for one week) including CAM in the patients with CAM-sensitive or CAM-resistant *H. pylori* [12–15]. We also used success rate of metronidazole included triple therapy (omeprazole 20 mg bid or lansoprazole 30 mg bid, amoxicillin 500 or 750 mg bid, metronidazole 500 mg in the morning and 250 mg in the evening or 250 mg tid for one week) in the patients with CAM-sensitive or CAM-resistant *H. pylori* [16]. In case of AMSGT, the *H. pylori* eradication rate of the 2nd regimen for the patients with CAM-resistant *H. pylori* was used [15]. The success rate of the third regimen (lansoprazole 30 mg bid, amoxicillin 750 mg bid, and sitafloxacin 100 mg bid for one week) for the patients who failed metronidazole-based triple therapy was also used [17].

Costs of each diagnostic procedure and *H. pylori* eradication regimens were derived from reimbursement of the Japanese governmental health insurance [18], and costs of gastric cancer treatments were derived from diagnosis procedure combination (DPC) by the Japanese government [19].

Our main outcome was a success rate of *H. pylori* eradication. Cost-effective thresholds, in other words willingness to pay (WTP), were estimated by treatment costs of preventable gastric cancer divided by the number needed to eradicate *H. pylori* infection.

Ford et al. [20] reported a pooled relative risk of 0.66 (95% confidence interval 0.46 to 0.95), and a number needed to eradicate *H. pylori* to prevent one patient of gastric cancer was as low as 15 for Chinese men, compared to 245 for US women.

Early gastric cancer is treated by endoscopic mucosal resection (EMR) or endoscopic submucosal dissection (ESD), and advanced gastric cancer is treated by laparoscopic or open gastrectomy. These DPC costs ranged from $2500 to $16000 [19].

At least, we may save $167 ($2500 × (1/15)) in high-prevalence areas or $10 ($2500 × (1/245)) in low-prevalence areas by successfully eradicating *H. pylori* infection in one patient. This means that WTP is at least $10 in low-prevalence areas and $167 in high-prevalence areas.

Expected values of cost and effectiveness were calculated for BC potentially for AMSGT and other six diagnostic strategies (RUT, histology, SHPAb, UBT, SHPAg, and UHPAb). Costs of each diagnostic method and each *H. pylori* eradication regimen were estimated from National Health Insurance data in Japan and expressed in US dollars at the exchange rate of 100 yen/US dollar (Table 1) [18, 19, 21]. We did not include cost of EGD as all patients in our model underwent EGD.

We first performed cost-effective analysis of base-case and calculated incremental cost-effective ratio (ICER) for comparing pairs of undominated diagnostic methods. Then, we conducted a one-way sensitivity analysis, focusing on the prevalence of CAM-resistant *H. pylori*, prevalence of *H. pylori* in the patients with AG, and the success rate of the 1st regimen for *H. pylori* to determine its threshold (Table 1).

We also performed a Monte Carlo simulation using range of uncertain probability in two scenarios of 0.4 or 0.45 of CAM-resistant *H. pylori* prevalence. All variables were assumed to follow a triangular distribution (Table 1). Ten thousand trials were conducted for simulation. We reported acceptability curve by a simulation of 10000 trials.

We used STATA® version14.1 (StataCorp, College Station, TX) for meta-analysis and TreeAge Pro® version 2016 (TreeAge Software, Inc., Williamstown, MA) for cost-effective analysis.

## 3. Results and Discussion

**Figure 1:**
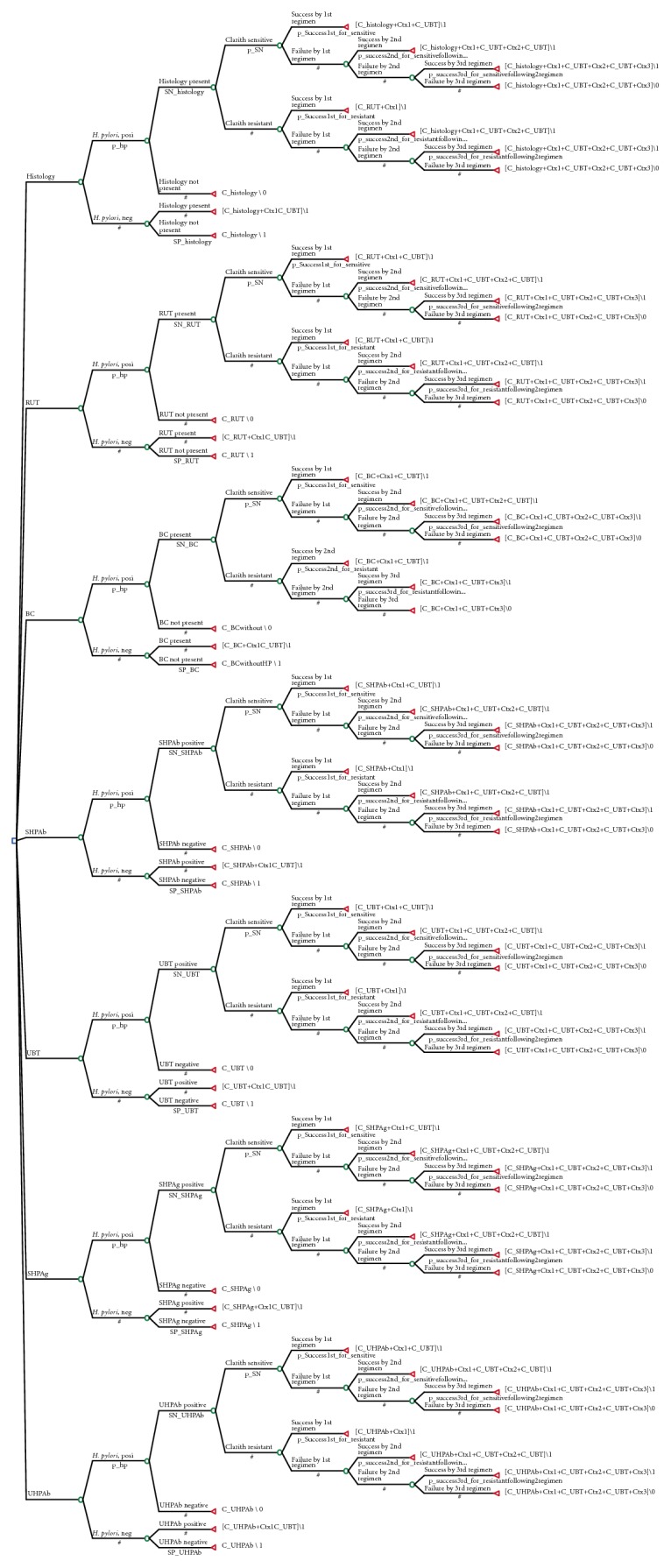
Decision tree. Decision tree was constructed on the assumption that treatment was selected after performing esophagogastroduodenoscopy in all patients.

A decision-analysis model starting at the point of diagnosing *H. pylori* infection during or just after EGD was constructed (Figure 1). Costs and probabilities used in the decision model are presented in Table 1.

Regarding diagnostic performance of BC, RUT, histology, UBT, SHPAb, and SHPAg, pooled values reported in past meta-analyses [22–25] were used. Our meta-analysis of 11 studies [26–36] about UHPAb showed that pooled sensitivity and specificity [95% CI] of UHPAb was 0.87 [0.72–0.94] (*I^2^*, 96%) and 0.94 [0.88–0.97] (*I^2^*, 84%), respectively. The publication bias was not significant (*P* = 0.62).

**Figure 2:**
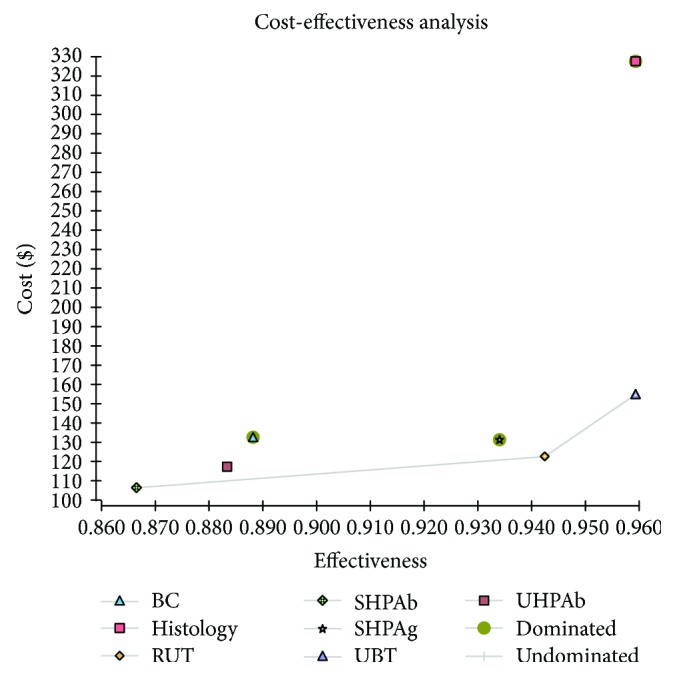
Cost-effectiveness graph. Cost-effectiveness analysis showed that histology, stool *H. pylori* antigen, and bacterial culture were absolutely and urine *H. pylori* antibody was weakly dominated by serum *H. pylori* IgG antibody, rapid urease test, and urea breath test.

With the current prevalence of CAM-resistant *H. pylori* of 30% [37], the most effective test for *H. pylori* diagnosis was UBT or histology, while the least effective test was SHPAb. Additionally, the most expensive test was histology, while the least expensive test was SHPAb. Histology, SHPAg, and BC were absolutely and UHPAb was weakly dominated by SHPAb, RUT, and UBT. Among the three undominated methods, the ICER of RUT versus SHPAb and UBT versus RUT was $214 and $1914, respectively. The *H. pylori* eradication success rate of SHPAb, RUT, and UBT was 0.87, 0.94, and 0.96, respectively (Figure 2).

**Figure 3:**
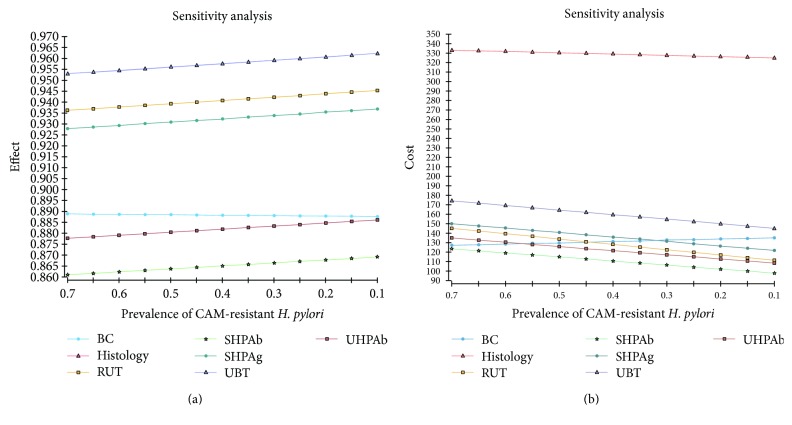
One-way sensitivity analysis. Sensitivity analysis using prevalence of clarithromycin- (CAM-) resistant *Helicobacter pylori (H. pylori)* showed that the order of effectiveness of seven diagnostic methods did not change between a CAM-resistant *H. pylori* prevalence of 0.1 and 0.7. The lines of histology and urea breath test were overlapped (a). In contrast, the cost of bacterial culture became equal to urine *H. pylori* antibody or rapid urease test or stool *H. pylori* antigen at between a CAM-resistant *H. pylori* prevalence of 0.3 and 0.58 (b).

**Figure 4:**
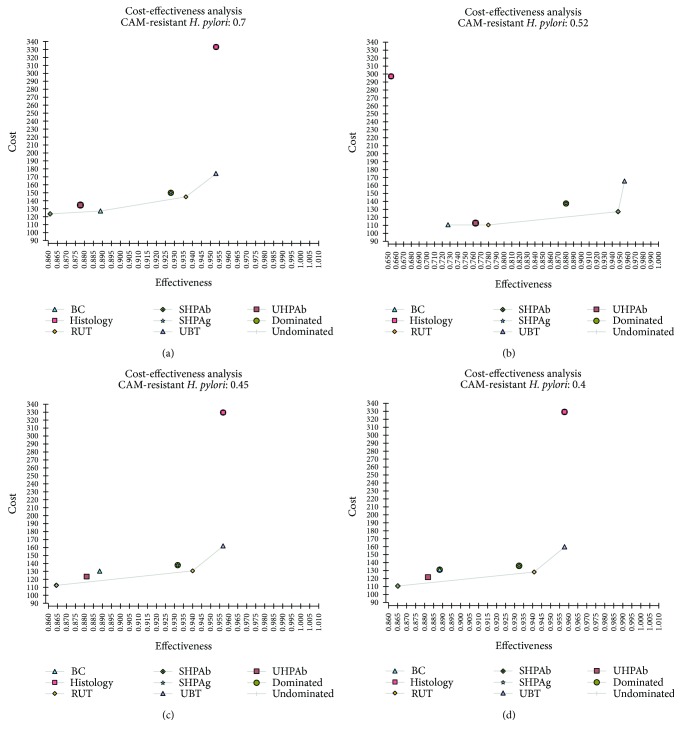
Cost-effectiveness plane of sensitivity analysis. If the prevalence of clarithromycin- (CAM-) resistant *H. pylori* was ≥0.45 (a), bacterial culture (BC) with antibiotics susceptibility testing was not dominated. However, if the prevalence of CAM-resistant *H. pylori* was ≤0.44, BC was dominated by serum *H. pylori* IgG antibody, rapid urease test, and urea breath test (b–d).

One-way sensitivity analysis with change of prevalence of CAM-resistant *H. pylori* was showed in Figures 3 and 4. In cost-effective plane, BC was dominated if the proportion of CAM-resistant *H. pylori* was less than or equal to 44%. However, if the proportion of CAM-resistant *H. pylori* was 45%, BC was not dominated. The *H. pylori* eradication success rate of SHPAb, UHPAb, BC, RUT, and UBT was 0.86, 0.88, 0.89, 0.94, and 0.96, respectively. The ICER of UHPAb versus SHPAb, BC versus UHPAb, RUT versus BC, and UBT versus RUT was $657, $932, $8, and $1853, respectively (Figure 4).

One-way sensitivity analyses using other two variables the prevalence of *H. pylori* in the patients with AG and *H. pylori* eradication success rate by the 1st regimen suggested that our results were insensitive for these two variables (Table 2).

**Figure 5:**
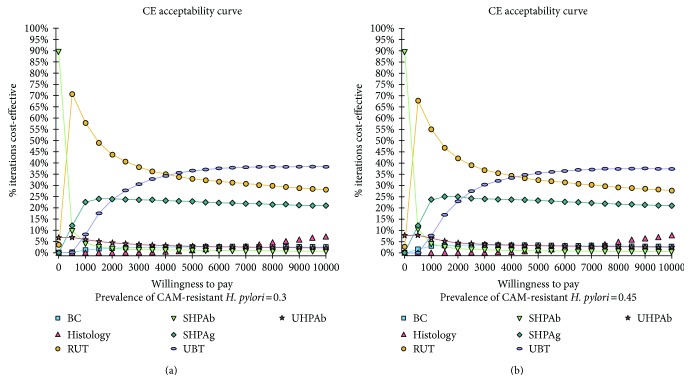
Acceptability curves using Monte Carlo simulation analysis with 0.3 (a) and 0.45 (b) of clarithromycin- (CAM-) resistant *Helicobacter pylori (H. pylori)*. Acceptability curve showed that serum *H. pylori* antibody or rapid urease test or urea breath test was an optimal diagnostic method depending on willingness to pay (WTP). Even if the prevalence of CAM-resistant *H. pylori* increased to 0.45, the probability that bacterial culture becomes an optimal method was low regardless of WTP.

In acceptability curves using Monte Carlo simulation with current (0.3) and increased (0.45) prevalence of CAM-resistant *H. pylori*, the optimal strategy was either SHPAb, RUT, or UBT (Figures 5(a) and 5(b)).

This is the first cost-effective analysis of *H. pylori* diagnostic methods mainly taking into account increasing prevalence of CAM-resistant *H. pylori*. First, our study showed that SHPAb, RUT, and UBT were undominated and RUT was the most cost-effective at the current prevalence of CAM-resistant *H. pylori* considering both their effectiveness and WTP. Second, although BC for AMSGT can be a suitable option if the proportion of CAM-resistant *H. pylori* increases to more than 45%, RUT was the most cost-effective as the effectiveness of BC was remarkably poorer than RUT and UBT. Third, although SHPAg was dominated in the base-case analysis, Monte-Carlo analyses showed that SHPAg was cost-effective in about 20% of trials if WTP was more than $1000. This would be caused by uncertainty of diagnostic performance of UBT and SHPAg.

Elwyn et al. [38] performed cost-effective analysis including three methods (SHPAb, SHPAg, and UBT) and concluded that UBT was dominated by SHPAg and the ICER of SHPAg versus SHPAb was €10. This study disregarded the three invasive tests, as well as UHPAb, all of which were included in our decision model. The cost of SHPAg ($33) is relatively more expensive than other diagnostic tests in Japan, and the above study used higher sensitivity and specificity data for SHPAg than those used in our model. The outcome of this study by Elwyn et al. was not the *H. pylori* eradication rate but the number of true outcomes. Additionally, authors did not discuss about WTP. These might be some of the reasons why SHPAg was not found to be cost-effective in our study. They followed “test and treat” policy without considering a referral to EGD, common in general practitioners' practice outside of Japan. In our decision model, we assumed that EGD would be performed prior to *H. pylori* testing in patients with or without dyspepsia.

According to the most recent guidelines for gastric cancer screening in Japan, EGD can be used not only as an opportunistic screening but also as a population-based screening tool [39]. It is anticipated that the number of asymptomatic individuals with the diagnosis of AG will increase and a cost-effective diagnostic tool for *H. pylori* infection is therefore needed. As such, the results of our study can be applied to choosing a diagnostic method for *H. pylori* infection mainly in the context of a screening population undergoing EGD.

Considering poor effectiveness of SHPAb and ICER of UBT versus RUT, RUT was the optimal choice for diagnosing *H. pylori* infection at the current CAM-resistant *H. pylori* prevalence.

If the prevalence of CAM-resistant *H. pylori* infection increases to 45%, BC becomes one of the options. Considering not only ICERs of UHPAb versus SHPAb ($657), BC versus UHPAb ($932), RUT versus BC ($8), and UBT versus RUT ($1853) but also poor effectiveness of SHPAb (0.86), UHPAb (0.88), and BC (0.89), RUT was again a preferred diagnostic method.

The CAM resistance of *H. pylori* was reported to be caused by mutations at two positions within 23S rRNA [40]. Okamura et al. [41] reported that the proportion of CAM-resistant *H. pylori* was significantly higher in younger groups. They also reported that the proportion of CAM-resistant *H. pylori* increased between 2000 and 2013, while the proportion of metronidazole-resistant *H. pylori* did not. We should clarify cost-effective diagnostic methods, anticipating future trends of increasing CAM-resistant *H. pylori* infections.

With recent understanding about pharmacokinetics of PPI, it has been reported that the efficacy of PPI included triple therapy is associated not only with antibiotics susceptibility but also with polymorphism of S-mephenytoin 4′-hydroxylase (CYP2C19) [42], a marker of rapid PPI metabolizers. We did not make our decision model considering this factor as the CYP2C19 test is not commercially available.

Our analysis has some limitations and strengths. First, we did not take into account possible adverse events from taking antibiotics or taking biopsy specimen for invasive tests, which we anticipate are very rare and not severe. Second, as treatment completion was assumed to be within 1 year, we did not consider the time needed until *H. pylori* eradication. Third, we did not take into account costs of several *H. pylori*-associated diseases except gastric cancer in estimating WTP. Fourth, our results can apply only for medical practice in Japan as our model assumes AG prevalence and standard *H. pylori* eradication regimen in Japan, both of which are different from western countries.

However, this is the first study to investigate the impact of increasing prevalence of CAM-resistant *H. pylori* infection from a cost-effectiveness perspective. In addition, we used the results of meta-analyses for all diagnostic methods' performance, which should be valid.

In conclusion, RUT was the most cost-effective diagnostic procedure given the present prevalence of CAM-resistant *H. pylori*. Although BC can be a cost-effective diagnostic method if the proportion of CAM-resistant *H. pylori* continues to increase to ≥45%, BC potentially for AMSGT will not be cost-effective due to its poor effectiveness.

## Figures and Tables

**Table 1:** Probabilities and costs.

Variable	Base case	References	Range for one-way sensitivity analysis	Range in Monte Carlo analysis
*Probabilities*				
Prevalence of *H. pylori* in AG	0.85	[11]	0.2–0.9	0.2–0.9
Proportion of CAM-resistant *H. pylori*	0.3	[37]	0.1–0.7	n. a.

*Sensitivity*				
Bacterial culture	0.87	[22]	n. a.	0.77–0.97
Rapid urease test	0.94	[22]	n. a.	0.84–1
Histology	0.96	[22]	n. a.	0.86–1
UBT	0.96	[23]	n. a.	0.86–1
Serum *H. pylori* IgG antibody	0.85	[24]	n. a.	0.75–0.95
Stool *H. pylori* antigen	0.93	[25]	n. a.	0.83–1
Urine *H. pylori* antibody	0.87	[26–36]	n. a.	0.77–0.97

*Specificity*				
Bacterial culture	0.96	[22]	n. a.	0.86–1
Rapid urease test	0.91	[22]	n. a.	0.81–1
Histology	0.77	[22]	n. a.	0.67–0.87
UBT	0.93	[23]	n. a.	0.83–1
Serum *H. pylori* IgG antibody	0.79	[24]	n. a.	0.69–0.89
Stool *H. pylori* antigen	0.96	[25]	n. a.	0.86–1
Urine *H. pylori* antibody	0.94	[26–36]	n. a.	0.84–1

*Success rate of eradication regimens*				
Success rate of 1st regimen for all	0.76	[12]	0.6–0.9	0.66–0.86
Success rate of 1st regimen for CAM-sensitive *H. pylori*	0.92	[13]	n. a.	0.82–1
Success rate of 1st regimen for CAM-resistant *H. pylori*	0.2	[14, 15]	n. a.	0.1–0.3
Success rate of 2nd regimen for CAM-resistant *H. pylori*	1	[15]	n. a.	0.9–1
Success rate of 2nd regimen for CAM-sensitive *H. pylori* after 1st regimen failure	0.9	[16]	n. a.	0.8–1
Success rate of 2nd regimen for CAM-resistant *H. pylori* after 1st regimen failure	0.9	[16]	n. a.	0.8–1
Success rate of 3rd regimen for CAM-sensitive *H. pylori* after 1st and 2nd regimen	0.67	[17]	n. a.	0.57–0.77
Success rate of 3rd regimen for CAM-resistant *H. pylori* after 1st and 2nd regimen	0.73	[17]	n. a.	0.63–0.83

*Costs*				
Diagnostics costs				
Bacterial culture with antibiotics sensitivity during EGD	$62	[18]	n. a.	n. a.
Bacterial culture only during EGD	$45	[18]	n. a.	n. a.
Rapid urease test during EGD	$20	[18]	n. a.	n. a.
Histology including immunohistochemistry during EGD	$234	[18]	n. a.	n. a.
UBT	$53	[18]	n. a.	n. a.
Serum *H. pylori* IgG antibody	$14	[18]	n. a.	n. a.
Stool *H. pylori* antigen	$33	[18]	n. a.	n. a.
Urine *H. pylori* antibody	$25	[18]	n. a.	n. a.

Antibiotics and administration costs				
LAC for one week (1st line)	$45	[21]	n. a.	n. a.
LAM for one week (2nd line)	$35	[21]	n. a.	n. a.
LAS for one week (3rd line)	$92	[21]	n. a.	n. a.

*H. pylori*, *Helicobacter pylori*; AG, atrophic gastritis; CAM, clarithromycin, UBT, urea breath test; EGD, esophagogastroduodenoscopy; LAC, lansoprazole 30 mg bid, amoxicillin 750 mg bid, and clarithromycin 200 mg bid; LAM, lansoprazole 30 mg bid, amoxicillin 750 mg bid, and metronidazole 500 mg bid; LAS, lansoprazole 30 mg bid, amoxicillin 500 mg bid, and sitafloxacin 100 mg bid; EMR, endoscopic mucosal resection; ESD, endoscopic submucosal dissection; n. a., not applicable.

^*^This range was not used in Monte Carlo analysis but in one-way sensitivity analysis.

**Table 2:** Results of one-way sensitivity analysis.

Variable	Base case	Threshold	Results of sensitivity analysis
Proportion of CAM-resistant *H. pylori*	0.3	0.45	BC was not dominated if CAM-resistant *H*. *pylori* ≥ 0.45.
Prevalence of *H. pylori* in AG	0.85	—	BC was dominated. RUT was the most cost-effective.
Success rate of 1st regimen for all	0.76	—	BC was dominated. RUT was the most cost-effective.

CAM, clarithromycin; *H. pylori*, *Helicobacter pylori*; AG, atrophic gastritis; BC, bacterial culture; RUT, rapid urease test.
